# Personal

**Published:** 1912-05

**Authors:** 


### PERSONAL

It gives us much pleasure to record two well deserved honors that have recently come to a life-long friend, Dr. Herbert Upham Williams of Buffalo. He has been made Acting Dean of the Medical Department of the University of Buffalo, vice Dr. M. D. Mann, resigned. On April 5, he was elected President of the American Association of Pathologists and Bacteriologists, at its meeting in Philadelphia. Dr. Williams is one of a comparatively small number of men who have found pathology broad enough to be used for the foundation of a life-work instead of a stepping stone. To such men we owe both the quantity of experience and the maturity of judgement so much needed in the establishment of a science. His work has been thorough rather than brilliant. Thus far, he has discovered no new disease nor disease germ. When he does, it will not be connected with tetrerythism.

---

Mrs. Lenora Wheeler Gay's Registry for Nurses has been removed to 723 Prospect Ave., Buffalo.

---

Dr. A. L. Borden of Gowanda has recovered $200 damages from the town of Evans, on account of a boulder in a road with which his automobile collided.

---

Dr. Frederick P. Hoyer of Tonawanda, the oldest Mason in the state, will celebrate his 90th birthday, May 9.

---

Dr. B. Ross Nairn of Buffalo, has moved to 512 Franklin St.

---

Dr. G. A. Neil of Buffalo, has moved from 1315 to 1278 Jefferson St.

---

Dr. Edgar Bieber has been appointed resident physician at the Ernest Wende Hospital, Buffalo, vice Dr. L. G. Lewis, resigned.

DR. EDITH R. HATCH has been elected a director of the City (Buffalo) Federation of Women's Clubs.

---

DR. C. E. BOWMAN of Alden returned from a southern trip, about the 1st of April.

---

DR. C. A. TYLOR of Alden has recently had a severe illness but is recovering.

---

DR. L. R. COLEGROVE of Elmira visied in Buffalo early in April.

---

DR. EDWARD CLARK of Buffalo spent Easter week at Old Point Comfort.

---

DR. JOHN O. ROE of Rochester has recently returned from a trip to Panama. He has promised a description of phases of the canal work that would interest our readers.

---

DR. NATHAN JACOBSON of Syracuse has returned from the Bermudas.

---

DR. FAYETTE H. PECK of Utica has returned from Europe.

---

DR. C. F. CHAFFE of Rochester has moved to 34 Gerard St.

---

DR. W. W. PLUMMER of Buffalo, read a paper on Fractures, illustrated with *x*-ray plates, before the Dunkirk and Fredonia Medical Society, April 10.

---

DR. E. R. LINKLATER of Buffalo, has recovered from a serious illness and is again at work.

---

DR. EVELINE P. BALLINTINE of Rochester State Hospital, has recently visited relatives in Warsaw.

---

DR. C. E. ABBOTT of Buffalo has been spending some time recently at the Chalfonte, Atlantic City.

---

DR. CURTISS N. JAMESON, Rochester, has removed his office to 330 West Avenue.

---

DR. ROBERT L. CARSON, Rochester, has removed his office to 54 Gibbs Street.

---

DR. FREDERICK W. SEYMOUR, Rochester, has removed to 355 East Ave.

---

DR. HARRY J. BRAYTON for four years a member of staff at the Raybrook State Tuberculosis Sanitarium, has been appointed

Medical officer of Iola Sanitarium, the County Tuberculosis Sanitarium of Monroe County.

---

Dr. J. F. W. Whitbeck of Rochester, has been elected president of the State Medical Society. The annual meeting will be held in Rochester in April next year.

